# Artificial Intelligence‐Based Detection of Colonic Angiodysplasia Can Improve Endoscopic Reporting Consistency

**DOI:** 10.1002/ueg2.70291

**Published:** 2026-09-11

**Authors:** Ioannis Kafetzis, Jana Sophia Theile, Stavros Dimitriadis, Jörg Albert, Wolfram Zoller, Alexander Meining, Alexander Hann

**Affiliations:** ^1^ Interventional and Experimental Endoscopy (InExEn) Department of Internal Medicine 2 University Hospital Würzburg Würzburg Germany; ^2^ School of Medicine Aristotle University of Thessaloniki Thessaloniki Greece; ^3^ Gastroenterology Private Practice Thessaloniki Greece; ^4^ Gastroenterology, Gastrointestinal Oncology, Hepatology and Infectious Diseases Klinikum Stuttgart Stuttgart Germany

**Keywords:** angiodysplasia, artificial intelligence, colonoscopy, computer vision, large language models, reporting

## Abstract

**Background and Study Aims:**

Colonic angiodysplasia (AD) is often found incidentally during colonoscopy and frequently omitted from reports, despite being a key cause of lower gastrointestinal bleeding. Artificial intelligence (AI) may improve reporting completeness. In this study, we developed and validated an AI system to support AD documentation.

**Methods:**

The system combines a computer vision (CV) model to detect AD in colonoscopy images and videos with a locally deployed open‐weight Large Language Model (LLM) to assess whether AD is mentioned in reports. It was externally validated on independent image datasets and prospectively collected videos and used to identify unreported AD cases.

**Results:**

The CV model was trained on 10,908 annotated images (986 with AD) from 993 colonoscopies. External validation of 3534 images from 239 procedures showed 94.5% accuracy, 84.4% sensitivity, and 95.9% specificity. The LLM detected AD documentation with 95.8% accuracy, 97.7% sensitivity, and 92.4% specificity. The combined system identified 88 cases where AD was visible but unreported. In 126 full‐length videos, 5 cases were physician‐reported, while the system detected 23 AD‐positive cases.

**Conclusions:**

Our AI system accurately detects AD and cross‐checks reports, improving documentation completeness and supporting standardized reporting while preserving privacy through local deployment.

## Key Summary

- Summarise the established knowledge on this subject
  ◦ Angiodysplasias are a common cause of lower gastrointestinal tract bleeding.
  ◦ Reporting of angiodysplasias is sparse and subjective.
  ◦ Artificial intelligence could support standardized reporting but has not so far been investigated for colonic angiodysplasias.
- What are the significant and/or new findings of this study?
  ◦ A computer vision model for identifying angiodysplasias in the colon was trained and externally validated.
  ◦ The AI is further validated with endoscopic videos to identify angiodysplasias.
  ◦ A system combining the trained AI with large language models can mitigate underreporting during colonoscopy.

## 1 Introduction

Angiodysplasia (AD) describes vascular abnormalities of the mucosal and submucosal veins and capillaries within the colonic wall [1]. Its development is strongly associated with advanced age and multimorbidity [2], and they represent a frequent cause of gastrointestinal bleeding in patients older than 60 years [3, 4]. The estimated prevalence of AD is approximately 2% among asymptomatic individuals [2], while ADs are identified in up to 40%–50% of patients presenting with hematochezia [5]. Colonoscopy remains the gold standard for the detection of colonic ADs.

Recent efforts have aimed to categorize colonic AD based on its endoscopic appearance [6]. However, these classifications lack correlation with clinically relevant outcomes such as bleeding risk. In routine practice, AD reporting remains inconsistent, mostly due to subjective assessment of clinical relevance by the endoscopist. This is further supported by the variability in terminology, where terms such as angiectasia [7] and vascular ectasia [8] are used to describe the same finding. Consequently, ADs may be underreported, potentially limiting appropriate clinical management and risk stratification.

Artificial intelligence (AI) has demonstrated substantial potential in gastrointestinal endoscopy [9, 10]. Initial applications focused on computer‐aided polyp detection during colonoscopy [11, 12], establishing a proof of concept for real‐time AI assistance. Subsequent developments have expanded to more advanced tasks, including polyp characterization [13, 14, 15] and size estimation [16, 17, 18]. These advances have led to standardized reporting of endoscopic findings and supported the development of AI‐based automated reporting systems [19, 20, 21], with emerging evidence of their feasibility in clinical practice [22]. However, interactive systems capable of analyzing endoscopy reports and actively assisting endoscopists by identifying and suggesting potentially missed findings are not yet available.

AI‐based detection of ADs has been investigated primarily in the small bowel, particularly in capsule endoscopy datasets [23, 24, 25]. In contrast, to our knowledge, no prior studies have evaluated AI‐based detection of colonic ADs using colonoscopy images and videos, nor the integration of such systems with automated reporting support.

In this study, we developed an AI system capable of detecting colonic ADs in endoscopic images and videos using a computer vision (CV) model and assessing whether ADs are documented in the corresponding examination reports using an open‐weight Large Language Model (LLM). When ADs are detected during the examination but not documented, the system generates a suggestion for the physician to describe the finding. The CV model was trained using a large single‐center dataset and externally validated on an independent dataset from a second center, including both still images and prospectively collected full‐length colonoscopy videos. Additionally, report analysis performance was evaluated by comparing the system output with clinical examination reports from the second center.

## 2 Materials and Methods

### 2.1 Study Design and Data Collection

This study utilized retrospectively collected image and report data from two centers in Germany, where data from center 1 were recorded between 1998 and 2020 and for center 2 between 2015 and 2021. Data from the first center was used for training, whereas center 2 was used for external image validation. Only examinations containing both the written report and photo‐documentation were included in the training and validation datasets. The written reports were then filtered for AD‐related terms such as “angio”, “ectasia”, “vascular”, and their variants in German.

Additionally, video recordings with their corresponding examination reports from center 2, recorded from October 2023 to March 2024 were utilized for external video validation of the proposed system. These data were recorded for a prospective trial ( [22], NCT06094270). Neither the training dataset nor the image‐validation dataset included images recorded using the endoscopic processor that was used to capture the video validation data.

### 2.2 AI‐Based Angiodysplasia Detection and Reporting

The developed system consists of two AIs: a computer vision (CV) model that identifies colonic AD in endoscopic images and an open‐weight Large Language Model (LLM) that assesses the examination report to identify if AD is mentioned.

The CV model followed the ResNet34 [26] architecture and was trained with data from center 1. It received an endoscopic image as input and assessed whether AD was present in it or not. The output was a value ranging from 0 to 1, indicating model confidence, with values close to 1 indicating the presence of AD and values close to 0 indicating the absence of it. The confidence threshold to separate positive and negative predictions was 0.5, following standard practice.

Evaluation of the examination report was performed using the open‐weight gpt‐oss [27] (OpenAI Inc., San Francisco, USA) LLM. The LLM was prompted to identify if the written report mentioned AD or not. The output of the LLM was either true or false, indicating whether AD was mentioned in the report or not. Furthermore, for positive predictions, the LLM provided the exact excerpt from the report based on which the assessment was made. The prompt for the LLM is described in the Supporting Information S1 (Prompt for the LLM).

### 2.3 Mitigation of Underreporting in Clinical Practice

**FIGURE 1:**
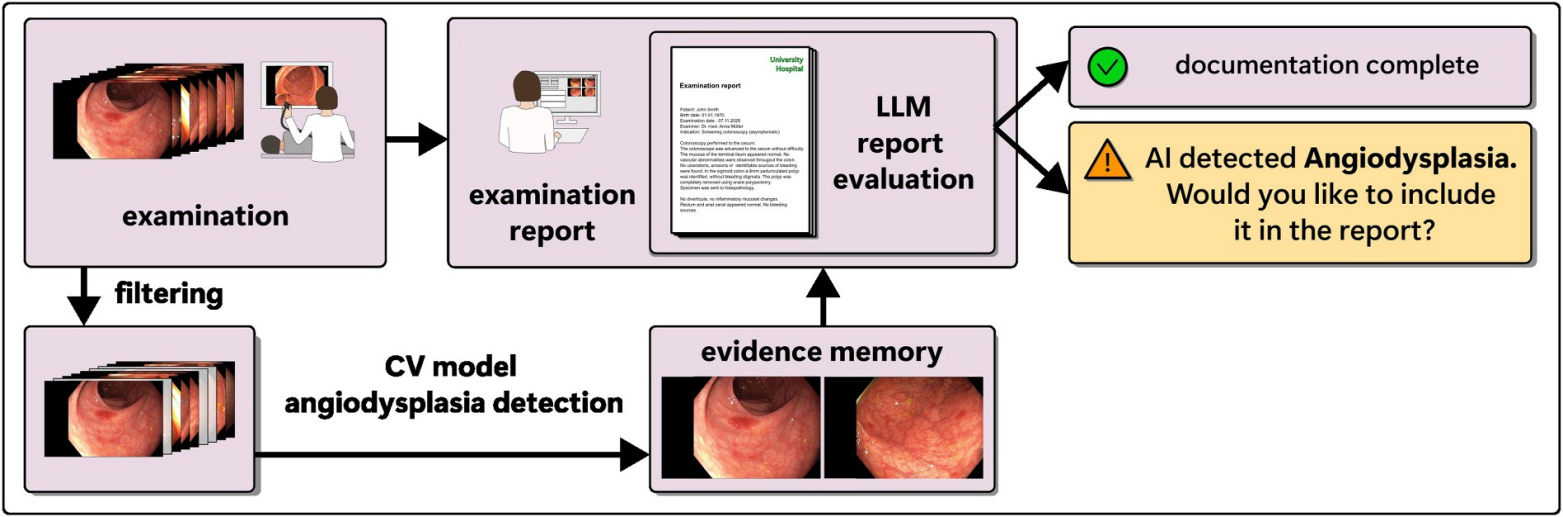
Visualization of the proposed AI system. The CV model identifies angiodysplasias in video frames and stores images as evidence. The LLM evaluates the examiner's written report and provides a notification if evidence of angiodysplasia is found but no direct mention in the examination text. AI, Artificial Intelligence; CV, Computer Vision; LLM, Large Language Model.

The proposed system can be introduced in clinical routine to support consistent reporting of AD. To this end, a multi‐classification AI, such as the one in [22], which has already been evaluated in clinical routine, is utilized to filter out irrelevant images, such as low‐quality images, images containing polyps, endoscopic instruments, blood, or wounds after polypectomy. Subsequently, the proposed CV model identifies any AD in the filtered images. For each identification of AD, the system records an image in its internal memory as evidence. At the end of the examination, once the examiner synthesizes the examination report, the LLM receives it to assess if AD is mentioned. In cases where the CV model identified AD, but the written report failed to mention it, the examiner receives a notification from the system to consider describing this finding, with the corresponding recorded images. Both AIs run locally to avoid any data privacy concerns. The proposed pipeline is visualized in Figure 1. External video validation was utilized to simulate implementation in clinical routines.

### 2.4 Evaluation and Statistics

The CV model was evaluated on the external image validation dataset in terms of accuracy, sensitivity, and specificity, along with bootstrapped 95% confidence intervals (95% CI). The explainability of model predictions was assessed using the GradCAM [28] method, which generates a heatmap indicating the parts of the input image with the highest influence on the model output. Furthermore, the CV model was used to identify examinations that contained photo‐documentation of AD but no AD‐related keywords in the report. Gold standard annotations were generated by an endoscopy resident together with an endoscopy expert. The expert adjudicated cases of diagnostic uncertainty and verified final lesion labeling. This annotation process was used for the training, external image validation, and video validation data.

The LLM was evaluated with examination reports from center 2 that contained either AD‐related terms or photo‐documentation. Evaluation metrics were accuracy, sensitivity, and specificity in identifying if AD was mentioned in the report. These reports were collected either based on keyword matching or AI predictions and were manually annotated by a physician to identify those that described AD. Furthermore, the LLM identified the keyword used in the report to describe AD. Examiner reports that mentioned AD were also screened to assess the size of the findings.

The entire system was evaluated with 126 prospectively collected videos from center 2. These videos were distinct from the test dataset. The CV model detected ADs in the video frames and the LLM identified if the finding was mentioned in the report. All identifications were validated via manual annotation through the collaboration of the endoscopy resident and gastroenterology expert. The system was evaluated for the number of examinations where AD was identified, the percentage increase of AD reporting, accuracy, and number of images recorded by the CV model. To determine the impact of physician experience in reporting AD, we considered a physician experienced if they have more than 5 years of experience in performing colonoscopy and young if they have less than that. Additionally, examination characteristics, including whether the withdrawal phase was over or under 8 minutes and the presence of interventions such as biopsies or polypectomies, were associated with the number of non‐reported AD cases. Associations were tested with the chi‐square test. The process of obtaining the entirety of the training and evaluation data is presented via flowcharts in Supporting Information S1: Figure 1s.

Additionally, data from external image validation sets were used to identify potential causes of underreporting. Statistical differences among different examination characteristic groups as well as among physicians performing the examinations were calculated using the chi‐square test. Examiners who conducted fewer than five examinations were excluded from this analysis.

### 2.5 Ethics

This study received approval from the medical ethics committee at Julius Maximilian University Würzburg (2025‐231‐dvhd). All patients enrolled in the prospective study signed informed consent prior to participation. The study was conducted in alignment with the Helsinki Declaration of 1964 and later versions.

## 3 Results

**FIGURE 2:**
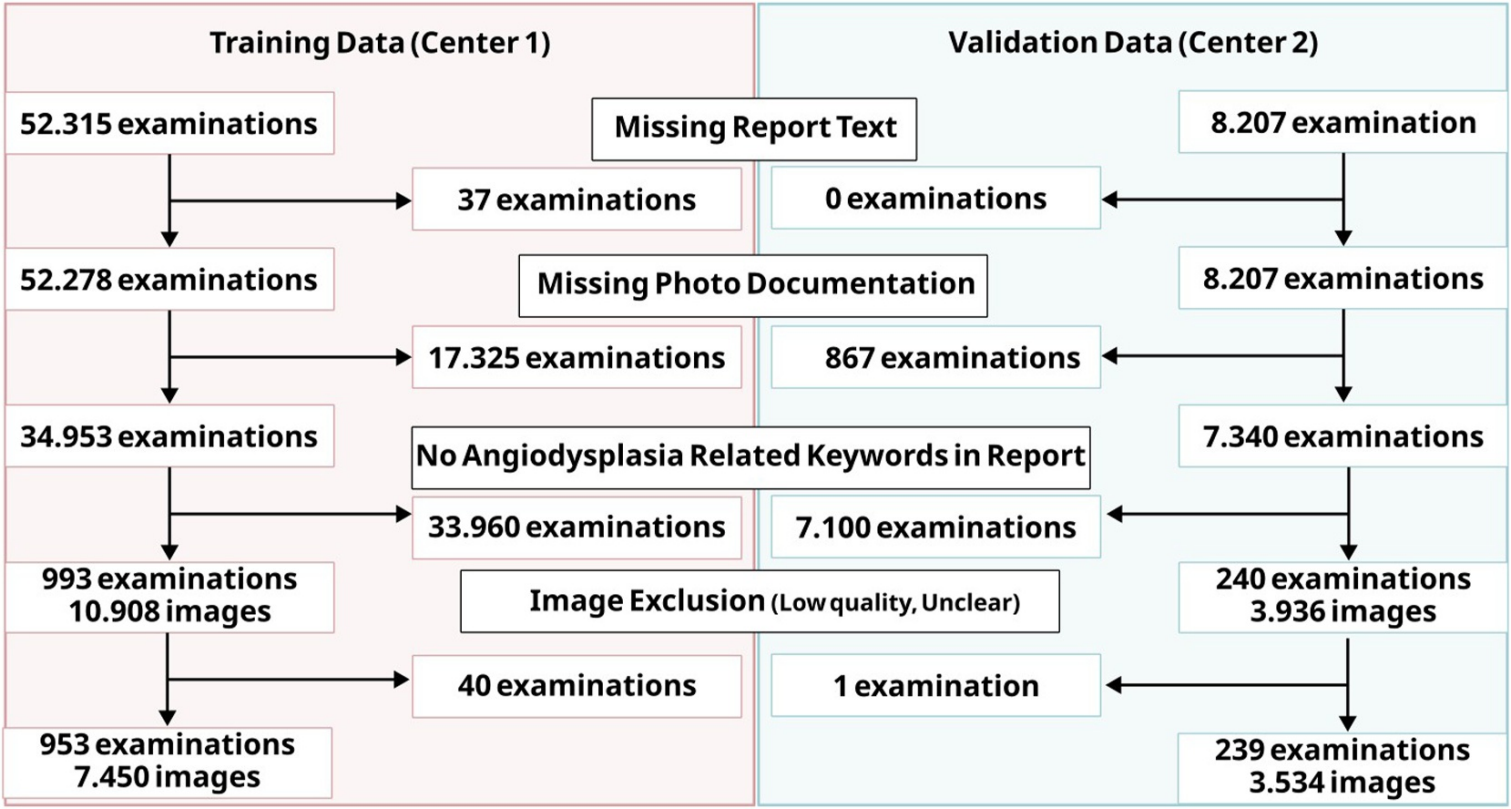
Data selection flowchart. The left column corresponds to data from the first center used for AI training. The column to the right corresponds to data from the second center used for external validation.

Out of a total of 10,908 images obtained from 993 examinations in center 1, 3458 were excluded due to duplication or low image quality. The remaining images were manually annotated to obtain gold standard labels. Of the remaining 7450 images that were used to train the CV model, 986 (13%) contained AD. Similarly, for the external validation dataset, 3936 images from 240 examinations were identified. Of these, 402 were excluded for the same reasons (duplicates, low quality). The final external image validation dataset comprised 3534 images from 239 examinations, which were again manually annotated. This resulted in 437 images from 175 examinations showing AD. The study data flowchart is presented in Figure 2. Video validation was performed on 126 examination recordings and their corresponding reports, recorded from December 2023 to March 2024. The median patient age was 64 years (Q1‐Q3: 56–71), with 64 patients (50.8%) being female.

**FIGURE 3:**
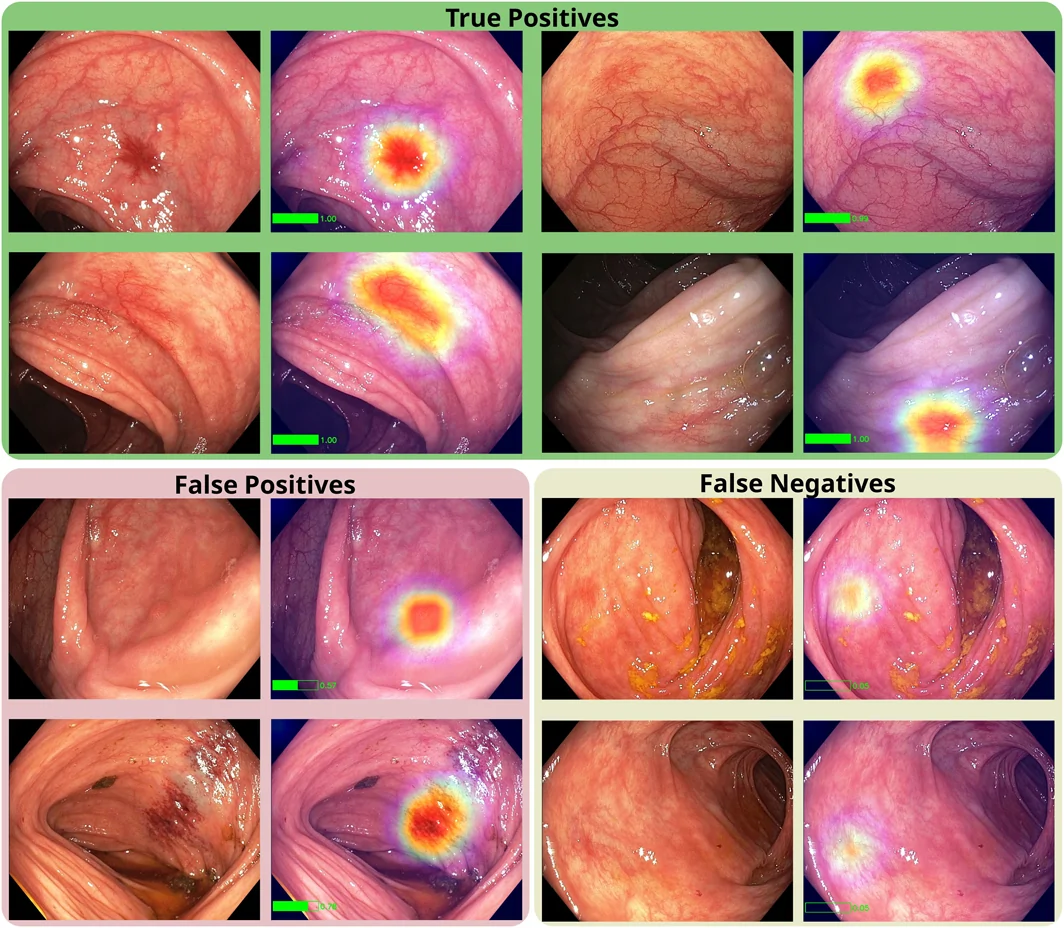
Explainability heatmaps for correct and erroneous AI predictions. The confidence of the AI is also displayed on the bottom left corner in the form of a green bar. AI, Artificial Intelligence.

For the external image validation using data from center 2, the CV model achieved an accuracy of 94.5% (95% CI: 93.7–95.2), sensitivity of 84.4% (95% CI: 81–87.9), and specificity of 95.9% (95% CI: 95.2–96.6). Correct and erroneous AI predictions were also evaluated using the GradCAM method to investigate the parts of the image having the highest impact on the AI decision. Heatmaps generated for correct and erroneous predictions are presented in Figure 3. Post hoc analysis showed that the AI correctly focused on the relevant regions of the images in most cases. In the cases of false negatives, although the AI focused on the correct parts of the image, the confidence did not reach high enough levels to be a positive prediction.

The performance of the open‐weight LLM to identify whether AD was described in the written report was evaluated using manually annotated data from 332 examinations, 214 (64.5%) of which were found to describe AD after manual annotation. The model achieved 95.8% accuracy, 97.7% sensitivity, and 92.4% specificity in identifying if AD was mentioned in the report. The LLM incorrectly flagged 9 reports as mentioning AD. In 7 of these (77.8%), the report mentioned vascular lesions in the rectum, which could also describe hemorrhoids or changes occurring as the result of radiation therapy. In the 5 cases where the LLM missed a true mention, it always extracted the correct evidence text from the report, but the final assessment was wrong.

Manual annotation of the 240 examinations selected through keywords for the external validation revealed that 204 (85.4%) of them mentioned AD as a finding. The LLM also correctly identified the term used to describe AD in 100% of positive predictions. Different terms used included angiodysplasia, angiectasia, vascular ectasia, telangiectasia, and phlebectasia in their German variants. The complete list of identified terms is described in Supporting Information S1: Table 1s. Manual review of the 214 reports from external image validation showed that 57 (27%) gave a numerical size estimate. In 85 (40%) qualitative terms such as tiny, small, or large, were used, of which 22 also gave a numerical value.

**FIGURE 4:**
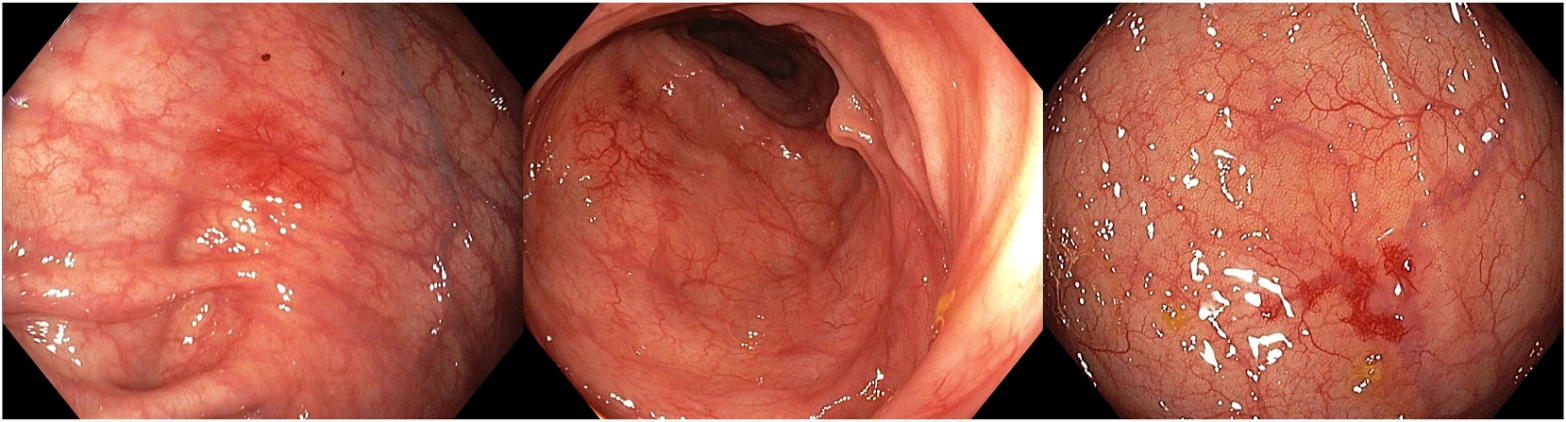
Example images of angiodysplasia that were recorded by physicians during the examination. In all these cases, angiodysplasia was not described as a finding in the examination report.

Furthermore, the CV model was used with images from examinations where AD was not described in the report to identify underreporting. The correctness was subsequently verified via manual annotation. Based on this analysis, 88 examinations containing AD in the photo‐documentation but not in the report were identified. With these results, the total number of examinations from the second center verified to have AD was 302. Examples of ADs that were photo‐documented but not included in the written report are presented in Figure 4.

Investigation of the examination characteristics revealed potential causes for underreporting of AD even in the presence of photo‐documentation (Table 1). AD was mentioned in 140 (75.7%) examinations where the patient was symptomatic and was neglected in 45 (24.3%) of these cases. Additionally, differences were observed for inpatient and outpatient settings. For inpatients, AD was reported in 69.4% of the cases, in contrast to 48.2% of the outpatient cases. The presence of complications resulted in no significant differences in the reporting of AD. For video validation, the manual annotation of the reports revealed that 5 (3.9%) of the reports described AD as a finding. The proposed system identified ADs in all these 5 examinations, and identified ADs in an additional 18 examinations, in which the finding had not been documented. At the examination level, the CV model achieved an accuracy of 67.5% and a specificity of 60.2%. The calculated sensitivity was 100%, which is a result of only evaluating examinations where the AI already had a positive image identification. Furthermore, the mean number of images selected by the CV model was 2.7 ± 2.1 per examination. The LLM correctly identified all 5 examinations describing AD and 3 additional false positives, achieving an accuracy of 97.6%. All five examinations that described AD were performed by young physicians.

**TABLE 1:** Association between clinical and procedural characteristics and the documentation of angiodysplasia in colonoscopy reports. Exclusion only occurred in cases where the corresponding information could not be determined in the written report.

	Total, *n* (%)	Angiodysplasia mentioned in report, *n* (%)	Angiodysplasia not mentioned in report, *n* (%)	*p*‐value
Indication				*p* < 0.0001
Symptomatic	185	140 (75.7%)	45 (24.3%)	
Surveillance	66	26 (39.4%)	40 (60.6%)	
Screening	77	39 (50.6%)	38 (49.4%)	
Symptoms				*p* = 0.027
Non‐bleeding	45	28 (62.2%)	17 (37.8%)	
Bleeding	140	112 (80.0%)	28 (20.0%)	
Complications				*p* = 0.947
Yes	87	55 (63.2%)	32 (36.8%)	
No	245	152 (62.0%)	93 (38.0%)	
Type of treatment				*p* < 0.001
Outpatient	114	55 (48.2%)	59 (51.8%)	
Inpatient	216	150 (69.4%)	66 (30.6%)	
ASA grade				*p* = 0.314
I	26	12 (46.2%)	14 (53.8%)	
II	216	139 (64.4%)	77 (35.6%)	
III	82	51 (62.2%)	31 (37.8%)	
IV	4	3 (75.0%)	1 (25.0%)	

Furthermore, the proportions of reported and unreported AD were analyzed for the different examiners performing the procedures. No significant differences were observed among the 10 examiners regarding the number of examinations with or without reported AD (*p* = 0.8542). The mean percentage of cases in which AD was not documented by the examiners was 29.8% ± 7.0. Examiner experience was not associated with the frequency of non‐reported ADs (*p* = 0.971). Withdrawal phase duration (*p* = 0.907) and presence of interventions (*p* = 0.177) also showed no significant association.

## 4 Discussion

This work introduces an AI‐based system that identifies AD in colonoscopy videos and supports its standardized reporting. The system consists of two components: a CV model that identifies AD in frames of endoscopic examination videos and a locally running LLM that assesses if the written report describes AD. During clinical routines, the AD detection AI runs in the background and records images of any identified ADs. The LLM evaluates the final examination report. When AD was detected during the examination but not mentioned in the report, the system prompts the physician to include the finding, providing the recorded images as evidence. Therefore, the proposed system can improve the frequency and standardization of reporting ADs, supporting future risk assessment and patient stratification. The CV model was trained on manually annotated image data from a university hospital and was externally evaluated on both video and images from a second center (center 2).

External video validation of 126 examination recordings simulated the applicability of the system in real clinical practice. All images recorded by the CV model, as well as the examination reports, were manually annotated to verify whether AD was present. Using our system, a total of 23 examinations containing AD were identified, with only 5 of them having the finding reported by the examiner in the final report. Therefore, usage of our system resulted in a significant increase in reporting the findings. Furthermore, the CV model selected three images on average as evidence, thus introducing minimal additional overhead for the physician from AI proposals.

During external image validation, the CV model achieved an accuracy of 94.5%. The high sensitivity of 84.4% combined with an excellent specificity of 95.9% highlights the strong classification performance of the AI. Furthermore, model predictions were evaluated using explainability heatmaps generated by GradCAM. Analysis of the generated heatmaps revealed that the AI focuses on the correct parts of the input image when assessing it. False positive findings of the model can be attributed to areas with high vascular complexity, such as those on the surface of adenomas or to iatrogenic mucosal injuries caused by the endoscope scratching the mucosa.

Inconsistencies between examiners when reporting AD were also identified in the image validation using our system. Our system identified a total of 88 examinations from center 2, where examiners failed to mention AD in the written report, although they had recorded images of the finding. Analysis of examination characteristics revealed potential confounders resulting in underreporting. It was observed that AD was reported in 75.7% of symptomatic patients, but only in 39.4% of cases where the indication was a surveillance colonoscopy and 50.6% for a screening colonoscopy. Additionally, AD was properly documented in 69.4% of inpatients, but in 48.2% of outpatient cases. Examiner experience, examination duration, and presence of interventions were available for the video validation dataset. None of these characteristics were associated with a significant difference in the frequency of non‐reported ADs. The limited sample size may have reduced the power of this analysis.

Furthermore, keyword matching failed to identify whether AD is described in the report in almost 15% of the cases, which further justifies the usage of LLMs. Furthermore, the LLM was always able to identify the term used by the examiner to describe the ADs, even in the presence of typographical errors. Therefore, the LLM could identify when synonyms are used and suggest replacing them with AD, which can support standardized reporting. Error analysis showed that most false positives came from the LLM failing to distinguish non‐specific mentions of vascular lesions in the rectum, where it was unclear if they referenced ADs, hemorrhoids, or changes due to radiation therapy. False negatives had no clear cause. Evidence extraction was correct in all these cases, but the final judgment was not. A self‐reasoning step or an agentic design may help reduce these errors. Size reporting was also inconsistent. Only 27% of reports gave a numerical value. When multiple ADs were present, examiners typically reported the size of the largest one. 40% of reports used qualitative descriptors like tiny or large, but only one in four of these also gave a number. This points to an additional layer of inconsistency in AD reporting, beyond whether the finding is mentioned at all.

Detection of AD using AI has been investigated in capsule endoscopy, where Noya et al. described a system leveraging color, texture, and morphology features that achieved 89.5% sensitivity and 96.8% specificity [29]. Furthermore, Mascarenhas et al. trained an AI model on 72 capsule examinations, reporting 88.5% sensitivity and 97.1% specificity [30], while Ribeiro et al. presented an AI that achieved 94.1% and 95.1%, respectively [24]. In multi‐center studies, Xie et al. evaluated human‐AI interaction in the reading of capsule endoscopy videos, showing that AI increased sensitivity to 96% from 75% while preserving a 100% specificity [31].

This work is also subject to limitations. First, the retrospective design introduces potential bias, as all training data were collected at a single institution. However, the model was externally validated on both images and videos and demonstrated strong discriminative performance, supporting its generalizability. Annotation was performed collaboratively, which did not allow formal assessment of interobserver agreement. Future studies should use independent dual annotation with agreement analysis to further validate the reference standard. In video validation, the CV model occasionally confused mucosal injury with AD. This limitation could be mitigated by enriching the training dataset with additional representative examples, thereby improving the model's discriminative capability. Furthermore, images showing active bleeding, which could potentially affect detection performance, were filtered out, as such cases would likely be identified and documented by the physician during clinical routine. In the image validation dataset, most underreported cases occurred in outpatient settings, possibly because these patients were asymptomatic and did not require endoscopic intervention. Nevertheless, accurate documentation and thorough medical history remain important, as prior knowledge of AD may facilitate faster identification of a potential bleeding source in future clinical scenarios. The performance of the CV model on video examinations showed lower accuracy and specificity compared with image validation. This was mainly driven by false positive AD identifications. Post hoc analysis showed that most false positives were mucosal injuries caused by the endoscope itself, which could resemble AD in appearance. Such images are rarely photo‐documented during routine endoscopy and were therefore nearly absent from the training and image validation datasets. Including such examples in future training data is expected to reduce false positives and improve video‐level performance. The sensitivity of 100% reflects the fact that only model‐positive frames were selected for annotation in the video cohort. The proposed AI system detects the presence of AD but does not assess lesion severity or bleeding risk. However, this work establishes a foundation for subsequent data collection and annotation efforts, which enable more detailed characterization and clinically meaningful classification of AD. Finally, this study does not evaluate the real‐world implementation of the proposed workflow. Questions regarding user acceptance, false‐alert burden, impact on reporting time, and influence on physician behavior remain open. A follow‐up study is planned to assess these aspects in clinical practice.

We believe that the integration of our AI system into routine clinical practice can enhance both the frequency and consistency of AD reporting while reducing examiner cognitive burden. This is supported by the strong discriminative performance of the CV model, the selection of only a limited number of representative images, and the minimal LLM feedback provided only prior to report finalization. By maintaining full physician control and delivering targeted, evidence‐based prompts, the system promotes standardized documentation without disrupting workflow. Furthermore, existing polyp sizing methods, for example using a water jet or a laser as a reference, could be applied for AD sizing. The LLM‐based reporting tool could also be extended to flag reports missing a numerical size estimate and prompt its inclusion.

## Funding

This work was supported by the Eva Mayr‐Stihl Foundation, Waiblingen, Germany.

## Ethics Statement

This study received approval from the medical ethics committee at Julius Maximilian University Würzburg (2025‐231‐dvhd).

## Consent

All patients enrolled in the prospective study signed informed consent prior to participation. The study was conducted in alignment with the Helsinki Declaration of 1964 and later versions.

## Conflicts of Interest

The authors declare no conflicts of interest.

## Supporting information


Supporting Information S1

## Data Availability

The data that support the findings of this study are available on request from the corresponding author. The data are not publicly available due to privacy or ethical restrictions.
