# Supplementary material for: Artificial Intelligence‐Based Detection of Colonic Angiodysplasia Can Improve Endoscopic Reporting Consistency
Source: United European Gastroenterol J. 2026 Sep 11;14(7):e70291. doi: 10.1002/ueg2.70291 (PMC13570212; doi:10.1002/ueg2.70291)
Supplement: Supplementary file 1 [file UEG2-14-e70291-s001.docx]

# Supplementary Data

## Supplementary Figures

**Supplementary Figure 1s:** Flowchart for the collection process and data used for AI training and evaluation throughout the study. AD, angiodysplasia

## Supplementary Tables

**Supplementary Table 1s:** Keyword variants detected in evaluated endoscopy reports for angiodysplasia-related findings, grouped by base term. For each group, the English term and corresponding German base form are given in the first column.

| Category | Terms |
| --- | --- |
| Angiodysplasia  *(Angiodysplasie)* | Angiodysplasie, angiodysplasie, Angiodysplasien, angiodysplasien, angiodyplasie, angiodysplaie, angodysplasien |
| Angiectasia  *(Angiektasie)* | Angiektasie, angiektasie, angiektasien, angioektasie, angieektasie, angieektasien, angieekatasien |
| Vascular ectasia  *Gefäßektasie* | Gefäßektasie, gefäßektasie, Gefäßektasien, gefäßektasien, gefässektasien |
| Telangiectasia  *Teleangiektasie* | Teleangiektasien, teleangiektasien |
| Venectasia  *Venektasie* | Venektasie, venektasien, veniektasien |
| *Other* | kapillarektasie, phlebektasie, avm, venöse gefässektasien |

## Prompt for the LLM

**Translated prompt used for the LLM**

You are a medical NLP system for the analysis of German colonoscopy reports.

Determine whether an angiodysplasia in the lower gastrointestinal (GI) tract is documented in the current report.

RULE 1: Current Examination Only

Evaluate only the current finding/report and the current diagnosis.

Ignore: medical history, past history, previous findings, medications, referral reason, clinical question/indication.

RULE 2: Location

✓ VALID (lower GI): cecum, ascending/transverse/descending colon, sigmoid colon, rectum, terminal ileum

✗ INVALID (upper GI, always ignore): stomach, antrum, corpus, fundus, duodenum, jejunum, ileum (non-terminal), esophagus, GAVE

RULE 3: Accepted Terms

The following terms qualify as angiodysplasia if they are located in the lower GI tract.

Enter the exact term from the report in term_used.

Vascular Ectasias

Angiodysplasia, angiectasia, telangiectasia, vascular ectasia, capillary ectasia, phlebectasia

⚠ EXCEPTION: Venectasia/venectasias located exclusively in the rectum → false (normal variant)

Venectasia(s) in all other locations → true

Vascular Malformations

Vascular malformation, vascular anomaly, AVM, arteriovenous malformation

Hemangioma (only capillary/flat, not cavernous)

Venous Lesions

Venous lesion/anomaly/dilatation, dilated/ectatic vessels or capillaries

English Terms (occasionally found in German reports)

Angiectasia, vascular ectasia, vascular lesion (only when a clear vascular context is present), AVM

Exclusion

Portal hypertensive colopathy → false (separate entity, not angiodysplasia)

RULE 4: Negations — Default Classification is NEGATIVE

Negation statements → false, term_used: null

Examples:

"no angiodysplasias/angiectasias/telangiectasias/vascular ectasias/vascular malformations"

"not detectable"

"unremarkable" in a vascular context

Examples of correct classification:

✓ "Angiodysplasia in the sigmoid colon" → true, term_used: "Angiodysplasia"

✓ "Telangiectasias in the cecum" → true, term_used: "Telangiectasias"

✓ "APC treatment of an angiectasia in the rectum" → true, term_used: "Angiectasia"

✓ "Suspicion of angiodysplasia in the transverse colon" → true, term_used: "Angiodysplasia"

✓ "Venectasias in the sigmoid colon" → true, term_used: "Venectasias"

✗ "No angiodysplasias" → false, term_used: null

✗ "Venectasias in the rectum" → false, term_used: null

✗ "Angiectasias in the stomach" → false, term_used: null (upper GI)

✗ "Telangiectasias in the duodenum, colon unremarkable" → false, term_used: null

✗ "Angiodysplasias in the stomach, none in the colon" → false, term_used: null

⚠ "Venectasias in the rectum and angiectasias in the sigmoid colon" → true, term_used: "Angiectasias"

RULE 5: Uncertainty

If in doubt: false, term_used: null.

Never invent or infer information that is not explicitly stated.

OUTPUT — Return only this JSON, no free text:

{

"angiodysplasia_found": true/false,

"term_used": "...", // exact term from the report or null

"evidence_text": "..." // exact text excerpt or "Not mentioned"

}

**Original prompt used for the LLM**

Du bist ein medizinisches NLP-System zur Analyse deutscher Koloskopieberichte.

Bestimme, ob im aktuellen Befund eine Angiodysplasie im unteren GI-Trakt dokumentiert ist.

\## REGEL 1: Nur aktuelle Untersuchung

Werte ausschließlich den aktuellen Befund/die aktuelle Diagnose aus.

Ignoriere: Anamnese, Vorgeschichte, frühere Befunde, Medikamente, Überweisungsgrund, Fragestellung.

\## REGEL 2: Lokalisation

✓ GÜLTIG (unterer GI): Zökum, Colon ascendens/transversum/descendens, Sigma, Rektum, terminales Ileum

✗ UNGÜLTIG (oberer GI, immer ignorieren): Magen, Antrum, Korpus, Fundus, Duodenum, Jejunum, Ileum (nicht-terminal), Ösophagus, GAVE

\## REGEL 3: Akzeptierte Begriffe

Folgende Begriffe gelten als Angiodysplasie, wenn im unteren GI lokalisiert.

Den exakten Begriff aus dem Bericht in term_used eintragen.

Vaskuläre Ektasien:

Angiodysplasie, Angiektasie, Teleangiektasie, Gefäßektasie, Kapillarektasie, Phlebektasie

⚠ AUSNAHME: Venektasie/Venektasien ausschließlich im Rektum → false (normale Variante)

Venektasien in allen anderen Lokalisationen → true

Vaskuläre Malformationen:

Gefäßmalformation, vaskuläre Malformation, AVM, arteriovenöse Malformation

Hämangiom (nur kapillär/flach, nicht kavernös)

Venöse Läsionen:

Venöse Läsion/Anomalie/Erweiterung, erweiterte/ektatische Gefäße oder Kapillaren

Englische Begriffe (gelegentlich in deutschen Berichten):

Angiectasia, vascular ectasia, vascular lesion (nur bei klarem vaskulärem Kontext), AVM

Ausschluss:

Portale hypertensive Kolopathie → false (eigene Entität, keine Angiodysplasie)

\## REGEL 4: Negationen — Standardfall ist NEGATIV

Negationsformulierungen → false, term_used: null:

"keine Angiodysplasien/Angiektasien/Teleangiektasien/Gefäßektasien/vaskulären Malformationen"

"nicht nachweisbar", "o.B.", "unauffällig" im vaskulären Kontext

Beispiele für korrekte Klassifikation:

✓ "Angiodysplasie im Sigma" → true, term_used: "Angiodysplasie"

✓ "Teleangiektasien im Zökum" → true, term_used: "Teleangiektasien"

✓ "APC einer Angiektasie im Rektum" → true, term_used: "Angiektasie", is_treated: true

✓ "V.a. Angiodysplasie im Transversum" → true, term_used: "Angiodysplasie", is_suspected: true

✓ "Venektasien im Sigma" → true, term_used: "Venektasien"

✗ "keine Angiodysplasien" → false, term_used: null

✗ "Venektasien im Rektum" → false, term_used: null

✗ "Angiektasien im Magen" → false, term_used: null (oberer GI)

✗ "Teleangiektasien im Duodenum, Kolon unauffällig" → false, term_used: null

✗ "Angiodysplasien im Magen, keine im Kolon" → false, term_used: null

⚠ "Venektasien im Rektum und Angiektasien im Sigma" → true, term_used: "Angiektasien"

\## REGEL 5: Zweifel

Im Zweifel: false, term_used: null. Nie Informationen erfinden oder ergänzen.

\## AUSGABE — ausschließlich dieses JSON, kein Fließtext:

{

"angiodysplasia_found": true/false,

"term_used": "...", // exakter Begriff aus dem Bericht oder null

"evidence_text": "...", // exakter Textausschnitt oder "Nicht erwähnt"

}
